# Childhood experiences and sleep problems: A cross-sectional study on the indirect relationship mediated by stress, resilience and anxiety

**DOI:** 10.1371/journal.pone.0299057

**Published:** 2024-03-20

**Authors:** Rola Ashour, Elizabeth J. Halstead, Stephen Mangar, Vanessa Khoo Qi Lin, Atiqah Azhari, Alessandro Carollo, Gianluca Esposito, Laura Threadgold, Dagmara Dimitriou

**Affiliations:** 1 Sleep Education and Research Laboratory, Psychology and Human Development, UCL-Institute of Education, London, United Kingdom; 2 Psychology and Human Development Department, UCL, IOE Faculty of Education and Society, London, United Kingdom; 3 Department of Clinical Oncology, Imperial College Healthcare NHS Trust, Charing Cross Hospital, London, United Kingdom; 4 Psychology Programme, School of Humanities and Behavioural Sciences, Singapore University of Social Sciences, Singapore, Singapore; 5 Department of Psychology and Cognitive Science, University of Trento, Rovereto, Italy; The University of Manchester, UNITED KINGDOM

## Abstract

**Background:**

Childhood experiences either adverse (ACE) or benevolent (BCE) can indirectly impact sleep quality in adult life, which in turn are modulated by the interplay of a variety of factors such as depression, anxiety, resilience and mental health problems.

**Methods:**

A cross-sectional observational study was conducted across the UK and the Middle Eastern countries during the COVID-pandemic on 405 participants. An online survey used a combination of questionnaires to assess ACE and BCEs. The following tools were then used to assess the contribution of resilience, stress, depression and anxiety respectively: Brief Resilience Scale (BRS), Perceived Stress Scale (PSS-10), Patient Health Questionnaire-2 (PHQ-2) and General Anxiety Disorder-2 (GAD-2) scale on childhood experiences. The extent of sleep disturbances experienced over a period of seven days was assessed using the PROMIS Sleep Disturbance Short-Form Tool. A serial-parallel mediation model was used to evaluate the impact of the mediators on childhood experiences and sleep quality.

**Results:**

Over 50% of the cohort were from Middle Eastern countries. Four or more BCEs were experienced by 94.3% of the cohort. In contrast, 67.9% of participants experienced at least one ACE before the age of 18 years, with moderate levels of stress, mild depression and anxiety were reported in 3.7%, 13% and 20% of participants respectively. Whilst 25.4% of participants reported having had four or more ACEs, with higher reports in the middle easter countries (32%). ACEs were found to correlate with sleep disturbance whilst BCEs showed an inverse correlation. The relationship between ACE and sleep disturbances was shown to be mediated by stress, and anxiety, but not by resilience or depression. Resilience and stress, and resilience and anxiety serially mediated the interaction between ACE and sleep disturbance. With regards to BCE, an inverse association with sleep disturbance was recorded with similar mediators of stress and anxiety observed.

**Conclusion:**

This study confirms the negative effects of ACEs, and the positive effects of BCEs on sleep in adulthood which are both mediated predominantly by psychological resilience, anxiety and stress. Strategies aimed at improving psychological resilience as well as addressing stress and anxiety may help improve sleep quality.

## 1. Introduction

Some of the main factors that can affect wellbeing and mental health are adverse childhood experiences [1]. Adverse Childhood Experiences (ACE) are potentially traumatic events that occur in childhood (up to 18 years of age) which includes experiencing violence, abuse or neglect, witnessing violence in the home or community and having a family member attempt or die by suicide [2]. ACEs have been associated with mental health issues such as anxiety, stress, and depression [1, 3] which can subsequently lead to attention deficits and poor academic attainment [4]. Several studies have shown that ACEs might predict sleep problems in later adulthood (e.g., [5–8]). However, this relationship appears to be partially mediated by depression and anxiety, as demonstrated in study carried out by Park et al. [9] on 737 adolescents in South Korea.

Resilience, which encompasses a person’s ability to adapt to the disturbing stimuli they experience by applying coping mechanisms, not only enables managing of stressful events, but also protects their mental health status and reduces the harm otherwise incurred [10]. It has thus been recognised that mental resilience has a protective function against ACEs and sleep disturbances [11, 12].

Benevolent Childhood Experiences (BCE) which pertain to positive experiences before the age of 18 years include the presence of beliefs that give comfort, at least one safe caregiver, enjoyment of school and a predictable home routine, are positively correlated with happiness. Specifically, BCEs have shown a significant positive effect on the resiliency of the individuals, increasing their capacity to do well in the face of adversity [13]. In contrast to ACEs, they are negatively associated with anxiety and depression [14].

The prevalence of ACEs may show geographical variation. In a recent study by AlHemyari et al. [15] the prevalence of ACEs was examined in the eastern region of Saudi Arabia. The authors reported some alarming outcomes, such as 82% of participants said that they were emotionally neglected and 76% were subjected to community violence. All these ACEs were associated with an increased risk of mental and physical illness [15].

This present study sought to examine the interplay between childhood experiences (ACE and BCE) and sleep outcomes in a mixed cohort of adult patients or varying ages and ethnic backgrounds from the UK and middle eastern countries. A serial-parallel mediation model was used to examine how resilience, stress, depression and anxiety may serve as mediators of the relationship between ACEs/BCEs and sleep.

## 2. Methods

### 2.1 Study design

This study used a cross-sectional online survey method. An online survey was conducted in Arabic and English using Qualtrics.

Ethical approval was obtained from the University College London, Institute of Education research ethics committee (Data Protection number: Z6364106 2021 07 22). The age criterium for this study was 16 years and above. All data were anonymous.

### 2.2 Participants

The study enrolled 492 participants, with 389 females (79.1%) and 91 males (18.5%). The average age was 34.7 (±14.4). Table 1 summarises the demographics of the cohort, including the percentages for uncollected data.

**Table 1:** Demographic characteristics of study population.

Demographic Variables	Value
**Gender [n (%)]**	
Female	389 (79.1%)
Male	91 (18.5%)
Missing	12 (2.4%)
**Ethnicity [n (%)]**	
White	215 (43.7%)
Arab	166 (33.7%)
Asian	35 (7.1%)
Mixed	24 (4.9%)
Other	8 (1.6%)
Missing	44 (8.9%)
**Country [n (%)]**	
UK	245 (49.8%)
Saudi Arabia	100 (20.3%)
Egypt	57 (11.6%)
Lebanon	12 (2.4%)
Pakistan	32 (6.5%)
Bahrain	3 (0.6%)
Missing	43 (8.7%)
**Education [n (%)]**	
High School / A-Level	120 (24.4%)
University Degree	277 (56.3%)
Other	95 (19.3%)
**Age **	
Minimum	16
Maximum	84
Mean (SD)	34.7 (±14.4)

## 3. Measures

### 3.1 Procedure

An online survey used a combination of questionnaires to assess childhood experiences and the contribution of resilience, stress, depression and anxiety respectively. Recruitment took place during the covid pandemic from April 2021 to July 2022 using multi-channel recruitment methods such as Twitter, Facebook, Instagram, WhatsApp. Individuals who expressed interest in participating in the study were sent a link. Before proceeding to the survey questions, the participants were asked to read the information sheet and to sign the informed consent form online. For participants younger than 18, the informed consent for caregivers was available.

### 3.2 Recording ACE/BCE

Participants were asked to record any cases in which they had gone through or sought any kind of treatment for health conditions, and to indicate whether mental conditions were associated with ACEs [16]. A list of common ACE-associated mental health conditions was presented to participants including depression, PTSD, obsessive-compulsive disorder (OCD) and anxiety [17–19]. Participants were given a checklist to tick all the corresponding boxes of the conditions they have experienced. An additional choice of “others: specify” followed by a textbox was also provided, where participants would report any other experiences that were not mentioned among the above-listed options.

*ACEs* were measured using the Adverse Childhood Experiences Questionnaire, which consists of 10-items [20]. It was used to assess one’s retrospective self-reported exposure to a series of stressful and potentially traumatic experiences occurring in childhood before the age of eighteen [21]. Participants were asked whether they were exposed to the following categories of ACEs: 1) Physical abuse; 2) Verbal abuse; 3) Sexual abuse; 4) Drug exposure; 5) Parental separation; 6) Domestic violence; 7) Suicidal or mentally ill household members; 8) Incarcerated household members; 9) Emotional neglect; 10) Physical neglect. Some examples of these questions include, “[d]id a parent or other adult in the household often … push, grab, slap, or throw something at you? or ever hit you so hard that you had marks or were injured?”. Participants were asked to choose one of the responses: “yes”, “no,” or “prefer not to say”. The ACE total scores were calculated by summing the number of ’yes’ responses for each participant, resulting in a range from 0 to 10. A scale from 0 to 10 was the basis range of ACE total scores, where high scores indicated the highest level of adversity exposure.

*BCEs* were measured using the 10-item Benevolent Childhood Experiences Scale [22]. This self-reported measure helped assess exposure to a range of positive early life experiences which were present before the age of eighteen, which include: 1) Predictable routines; 2) A good friend; 3) Beliefs giving comfort; 4) A teacher who cared; 5) An adult in whom the participant could confide; 6) Opportunities to have a good time; 7) A positive self-image; 8) A liking for school; 9) A safe parent/caregiver; 10) Good neighbours. Responses were recorded using a dichotomised response matter; 1 equates as ‘yes’ while 0 equates ‘no’. A scale from 0 to 10 was the basis range of BCE total scores, where high scores indicated the highest level of positive early childhood experiences.

### 3.3 Questionnaires

#### 3.3.1 Brief Resilience Scale

Resilience was measured using the Brief Resilience Scale [23]. Questions such as *“In the last month*, *how often have you felt that things were going your way*?*”* were meant to assess the participants’ levels of resilience. Participants were given an overall score for their resilience based on these responses: “never,” “almost never,” “sometimes,” “fairly often,” “very often,” and “prefer not to say” while each was given a numeric value. Total scores range from 0–25. The scale demonstrated excellent internal reliability with a Cronbach’s alpha of 0.918.

#### 3.3.2 Stress

The Perceived Stress Scale (PSS-10) [24] consists of 10 items to measure the degree to which one perceives aspects of one’s life as uncontrollable, unpredictable and overloading. Perceived Stress Scale scores range from 0 to 40. Participants were asked to respond to each question on a 5-point Likert scale ranging from 0 = never, 1 = almost never, 2 = sometimes 3 = fairly often to 4 = very often. PSS-10 scores were obtained by reversing responses to the four positively stated items (4,5,7,8). The PSS-10 rated low stress (0–13) moderate stress (14–26) high perceived stress (27–40). The PSS-10 revealed good internal reliability with a Cronbach’s alpha of 0.678.

#### 3.3.3 Anxiety

General Anxiety Disorder-2 (GAD-2) Scale [25] is a brief two-item screening tool used to assess symptom frequencies of Generalised Anxiety Disorder over a span of two weeks. Responses for the questions were presented in the form of Likert scales ranging from “0” (none) to “3” (almost daily). It reflects all the Diagnostic and Statistical Manual of Mental Disorders, Fourth Edition (DSM-IV) symptom criteria for Generalised Anxiety Disorder. For each item, the response option is not all = 0, several days = 1, more than half the days = 2 and nearly every day = 3. The GAD-2 scale score ranges from 0 to 6. A score of ≥ 3 was indicative of clinically significant anxiety. The GAD-2 showed excellent internal reliability with a Cronbach’s alpha of 0.883.

#### 3.3.4 Depression

Depression was measured using the Patient Health Questionnaire-2 (PHQ-2) scale (The PHQ-2 includes the first two items of the PHQ-9 [26]. The tool assessed the frequency of depressed moods over two weeks and is used as a screening tool for depression. The PHQ-2 inquires about the frequency of a depressed mood and anhedonia over the past 2 weeks, scoring each as 0 ("not at all") to 3 ("nearly every day"). Similar to the General Anxiety Disorder-2 scale, participants responded to the PHQ-2 using two Likert-scales ranging from “0” (none) to “3” (almost daily), and higher scores indicated a higher frequency of symptoms of depression. The scale reflected good internal reliability with a Cronbach’s alpha of 0.767.

#### 3.3.5 Sleep

The extent of sleep disturbances experienced over a period of seven days was assessed using the PROMIS Sleep Disturbance Short-Form Tool [27]. The scale includes four measuring items: Sleep quality, Sleep refreshment, Sleep problems *and* Difficulty falling asleep. Each was scaled between 1–5. A total sleep disturbance score was calculated for each participant by reverse coding two items and subsequently cumulating scores across the four components. The scores ranged from 0–20, with higher scores indicating greater sleep disturbance. This scale demonstrated excellent internal reliability with a Cronbach’s alpha of 0.89.

A serial-parallel mediation model was used to evaluate the impact of the mediators on childhood experiences and sleep quality.

### 3.4 Analytical plan

**Fig 1:**
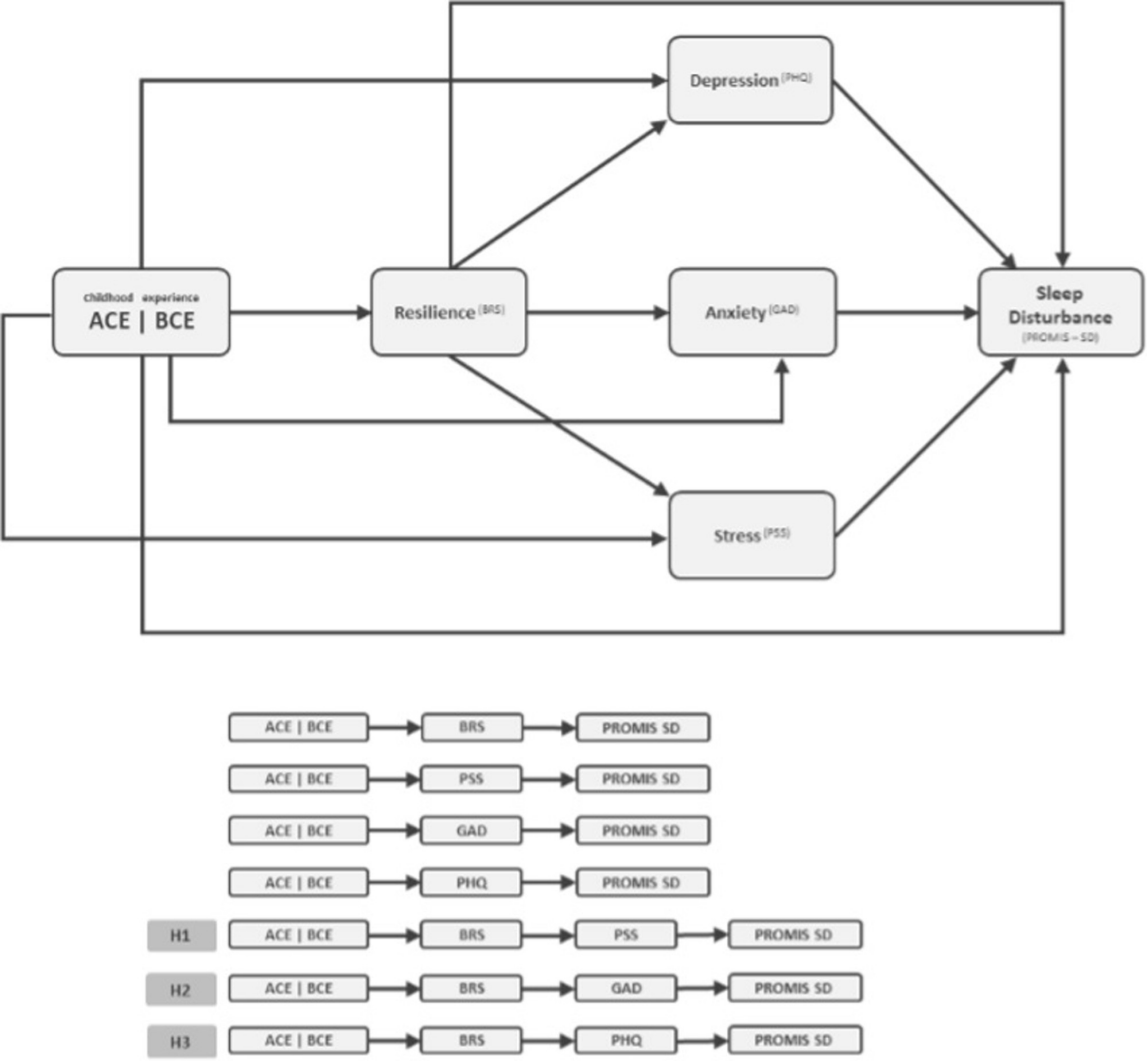
Conceptual serial-parallel mediation model diagram to investigate the effect of adverse and benevolent childhood experiences on sleep disturbance.

The survey contained a series of validated tools which assessed the resilience of the participants in terms of the indicators of stress, anxiety, depression, and crucial life events. In this study, there were two main predictor variables: ACE and BCE. ACE and BCE were mediated by resilience and mental health measures (depression, stress and anxiety). The outcome variable in this study is sleep disturbance. In Fig 1, the conceptual diagram is illustrated based on the outlined research hypotheses. The diagram demonstrates a preliminary serial-parallel mediation model with ACE or BCE as predictor variables, Sleep disturbance (PROMIS-SD) as the outcome variable, and resilience (BRS), depression (PHQ), anxiety (GAD) and stress (PSS) as mediators (Fig 1).

A priori power analysis was conducted to estimate the required sample size to detect a meaningful effect. Previous studies showed that the effect size can fluctuate between small to large and they also used medium effect size to calculate their required sample size. Due to the complexity of the proposed model, a conservative option ‐ the ’Linear multiple regression: Fixed model, R2 deviation from zero’ ‐ in G*Power software has been used. With a medium effect size of 0.15, in order to achieve 95% power at a significance level of 0.05 a minimum sample size of 160 is reported to be required.

A Little’s Missing Completely at Random (MCAR) test was employed to assess the nature and pattern of missing data, resulting in a χ^2^ (7) = 4.022, p = 0.77, suggesting that the missing data was independent of both the observed and the unobserved data. As such a listwise deletion method for statistical analysis was used to exclude the participants with missing values, resulting in a final sample of 405 participants for the analysis.

Means, standard deviations, minimum and maximum values for each of the seven variables are calculated. In order to report the prevalence of self-reported mental health issues and their severity, these measures were treated as categorical variables based on their score classification. It is important to note, that mental health measures were treated as continuous variables in the analysis of the conceptual model. Pearson’s two-tailed r correlation test was also conducted to examine the level of association between different variables. Normality of the data was also investigated using Shapiro-Wilk test and histograms. Due to the non-normality of the data, non-parametric tests, including the Kruskal-Wallis H test and Mann-Whitney U test were conducted to investigate whether there is a significant difference in variable scores among ethnicities, gender and education groups. Pearson’s r correlation test was also conducted to examine whether age is significantly associated with each of the seven variables. Each demographic variable, which shows a significant association with mental health measures or resilience, is treated as a controlling variable.

Finally, to analyse the serial-parallel mediation model proposed in Fig 1, “PROCESS” Macro of Hayes was used. The model number used was Model 81 with 95% confidence interval and bootstrapping (5000 samples). ACE or BCE were inputted as X (predictor variable), sleep disturbance was treated as Y (outcome variable), while resilience and mental health measures were inputted as mediators. Age, ethnicity, country and education were inputted as covariates. All calculations and analyses were also conducted by the SPSS V.26 software (Windows. Armonk, NY: IBM Corp, USA).

## 4 Results

### 4.1 Descriptive statistics

Preliminary analysis of the collected data indicated that 67.9% of participants experienced at least one ACE before the age of 18 years, while 25.4% of participants reported having had four or more ACEs. It is worth noting that the percentage of people reported to have experienced at least one ACE before the age of 18 is likely to be an underestimation. Only one individual reported having no BCE, while 94.3% of participants reported four or more than four BCEs. On the mental health measures, 96.3% of participants reported none or mild symptoms of stress, while 3.7% of participants reported moderate levels of perceived stress. 13% of participants reported being mildly depressed, while 87.2% of participants showed no or minimal signs of depression. The prevalence of anxiety was higher than other mental health issues. Table 2 depicts the level of association between different variables. The results of correlation test revealed a statistically significant (p < 0.001) association between each of the seven variables used in the mediation model. Using Cohen’s effect size thresholds, correlation’s data indicate that ACE and BCE have a high negative correlation (r = -0.501 p < 0.001). The strength of association between ACE and mental health measures is small but significant, while a medium level of strength of association between BCE and mental health measures and resilience was observed. In addition, the results revealed a medium to large correlation between resilience and sleep disturbance, depression, anxiety and stress. The strongest association of sleep disturbance was observed with stress (r = 0.395, p < 0.001). On the mental health measures, stress showed the highest negative correlation with resilience (r = -0.552, p < 0.001).

**Table 2:** Correlation matrix with predictor, mediators and outcome variables (* correlation is significant at the 0.05 level (2-talied); ** correlation is significant at the 0.01 level (2-tailed)).

Variables	Mean	SD	1	2	3	4	5	6	7
**1. ACE**	**2.2**	2.30	1						
**2. BCE**	**7.99**	2.24	-.501**	1					
**3. BRS**	**18.81**	4.91	-.156**	.344**	1				
**4. PSS**	**7.35**	3.42	.225**	-.312**	-.552**	1			
**5. GAD**	**2.63**	1.94	.241**	-.323**	-.493**	.617**	1		
**6. PHQ**	**2.01**	1.86	.238**	-.349**	-.470**	.647**	.686**	1	
**7. SLEEP**	**11.12**	4.29	.210**	-.266**	-.251**	.395**	.393**	.366**	1

Non-parametric tests (Kruskal-Wallis H test, Mann-Whitney U test) were used to examine whether there are significant differences in the scores of different variables based on the demographic groups (gender, ethnicity, education and country). Age was used as a continuous variable, and its correlation with other variables was investigated using two-tailed Pearson’s r correlation test. The results indicate that age had a small but statistically significant correlation with resilience, stress, anxiety, depression and sleep disturbance. Bivariate analysis of mental health issues and country of residence showed that the score of anxiety, stress and depression were significantly different depending on the country of residence. The score of mental health issues and resilience was also shown to differ significantly based on the education of participants. However, no significant difference in scores of mental health measures and resilience were observed between males and females. Table 3 reports the results from the bivariate analysis examining the effect of demographic factors on mental health measures and resilience.

**Table 3:** Bivariate analysis examining the effect of demographic factors on mental health measures and resilience. Bold numbers: Statistically significant. The numbers represent the main statistic (H for Kruskal-Wallis H test; U for Mann-Whitney U test; r for Pearson’s r correlation) and the associated p value in brackets.

Variables	Gender [U (*p*)]	Ethnicity [H (*p*)]	Education [H (*p*)]	Country [H (*p*)]	Age [r (*p*)]
ACE	17,110 (0.613)	7.998 (0.092)	0.299 (0.861)	**11.928 (0.036)**	0.081 (0.079)
BCE	16,534 (0.315)	8.431 (0.077)	1.823 (0.402)	**18.546 (0.002)**	-0.012 (0.801)
BRS	15,600 (0.077)	2.032 (0.730)	**10.361 (0.006)**	5.127 (0.401)	**0.168 (0.000)**
PSS	17,206 (0.678)	**10.296 (0.036)**	**12.395 (0.002)**	**25.031 (0.000)**	**-0.286 (0.000)**
GAD	16,064 (0.162)	**16.798 (0.002)**	**28.186 (0.001)**	**21.929 (0.001)**	**-0.296 (0.001)**
PHQ	17,630 (0.952)	**40.220 (0.001)**	**44.967 (0.001)**	**67.472 (0.001)**	**-0.355 (0.001)**
Sleep	17,119 (0.625)	5.088 (0.278)	4.178 (0.124)	3.066 (0.690)	**-0.153 (0.001)**

### 4.2 Serial-parallel mediation model analysis

As shown in Fig 1, the model consists of one predictor variable (ACE or BCE), and four mediators to analyse how adverse or benevolent childhood experiences predict sleep. Resilience and other mental health measures (depression, stress and anxiety) were placed serially in the model, while the mental health measures were parallel to each other. The outcome variable was sleep disturbances, age, ethnicity, education and country of residence were included as covariances.

Table 4 shows significant negative interaction between ACEs with resilience (b = -0.367, t = -3.390, p < 0.001). In addition, ACEs were shown to affect depression (b = 0.147, t = 4.416, p < 0.001), anxiety (b = 0.136, t = 3.817, p < 0.001) and stress (b = 0.236, t = 3.824, p < 0.001), directly and positively. In contrast, resilience had significant negative interaction with depression (b = -0.128, t = -8.439, p < 0.001), anxiety (b = -0.160, t = -9.760, p < 0.001), and stress (b = -0.330, t = -11.654, p < 0.001). ACEs (b = 0.133, t = 1.546, p = 0.123), resilience (b = 0.031, t = 0.686, p = 0.493) and depression (b = 0.301, t = 1.949, p = 0.052) have no direct effect on sleep disturbance, suggesting that mediation effects could potentially exist.

**Table 4:** Serial–parallel mediation model with adverse childhood experience as predictor variable. Abbreviations: ACE = Adverse childhood experience, BRS = Resilience, PHQ = Depression, GAD = Anxiety, PSS = Stress, SD (PROMIS-SD) = Sleep disturbance ‐ (Bold numbers: Statistically significant). Total effect model, direct effects and indirect effects are reported.

*Model*: *Adverse Childhood experience (ACE) as predictor variable*							
**Total Effect Model**		**b**	**t**	**p**	**95% CI**		**Conclusion**
**ACE ‐ SD**		**0.376**	**4.11**	**< 0.001**	**0.196**	**0.555**	
*R*^*2*^ *= 0*.*08 | F (399) = 6*.*850 | p < 0*.*001*							
**Direct effects**		**b**	**t**	**p**	**95% CI**		
**ACE ‐ BRS**		**-0.367**	**-3.390**	**< 0.001**	**-0.579**	**-0.154**	ü
ACE ‐ SD		0.133	1.546	0.123	-0.036	0.303	û
**ACE ‐ PHQ**		**0.147**	**4.416**	**< 0.001**	**0.082**	**0.213**	ü
**ACE ‐ GAD**		**0.136**	**3.817**	**< 0.001**	**0.066**	**0.207**	ü
**ACE ‐ PSS**		**0.236**	**3.824**	**< 0.001**	**0.115**	**0.357**	ü
BRS ‐ SD		0.031	0.686	0.493	-0.058	0.120	û
**BRS ‐ PHQ**		**-0.128**	**-8.439**	**< 0.001**	**-0.160**	**-0.098**	ü
**BRS ‐ GAD**		**-0.160**	**-9.760**	**< 0.001**	**-0.191**	**-0.128**	ü
**BRS ‐ PSS**		**-0.330**	**-11.654**	**< 0.001**	**-0.384**	**-0.273**	ü
PHQ ‐ SD		0.301	1.949	0.052	-0.003	0.606	û
**GAD ‐ SD**		**0.365**	**2.605**	**< 0.05**	**0.090**	**0.641**	ü
**PSS ‐ SD**		**0.349**	**4.401**	**< 0.001**	**0.193**	**0.504**	ü
**No.**	**Indirect effects**	**b**	**Boot SE**		**Boot 95% CI**		
ACE ‐ BRS ‐ SD		-0.011	0.019		-0.055	0.023	û
**ACE ‐ PSS ‐ SD**		**0.082**	**0.030**		**0.032**	**0.150**	ü
**ACE ‐ GAD ‐ SD**		**0.050**	**0.025**		**0.008**	**0.105**	ü
ACE ‐ PHQ ‐ SD		0.044	0.028		-0.001	0.107	û
**H1**	**ACE ‐ BRS ‐ PSS ‐ SD**	**0.042**	**0.017**		**0.014**	**0.080**	ü
**H2**	**ACE ‐ BRS ‐ GAD ‐ SD**	**0.021**	**0.012**		**0.003**	**0.049**	ü
**H3**	ACE ‐ BRS ‐ PHQ ‐ SD	0.014	0.010		-0.001	0.036	û

**Fig 2:**
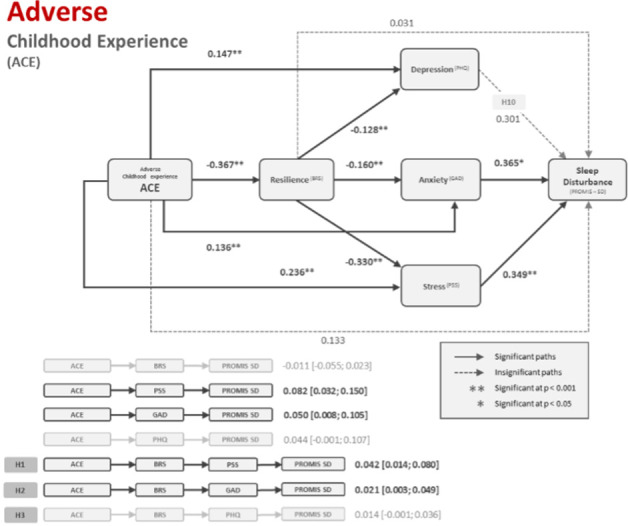
Flow diagram of serial–parallel mediation model with ACE as predictor variable.

Analysing the model for indirect effects reveal that resilience alone does not act as a mediator between ACE and sleep disturbance (b = -0.011, 95% CI [-0.055, 0.023]). The relationship between ACE and sleep disturbance was shown to be mediated by stress (b = 0.082, 95% CI [0.032, 0.150]) and anxiety (b = 0.050, 95% CI [0.008, 0.105]). No significant interaction was found between depression and sleep disturbance; hence, depression could not be observed as a mediator in this model. Resilience and stress were shown to serially mediate the interaction between ACE and sleep disturbance (b = 0.042, 95% CI [0.014, 0.080]). In addition, resilience and anxiety serially mediated the interaction between ACE and sleep disturbance (b = 0.021, 95% CI [0.003, 0.049]). A summary of the results can be found in the diagram presented in Fig 2.

**Fig 3:**
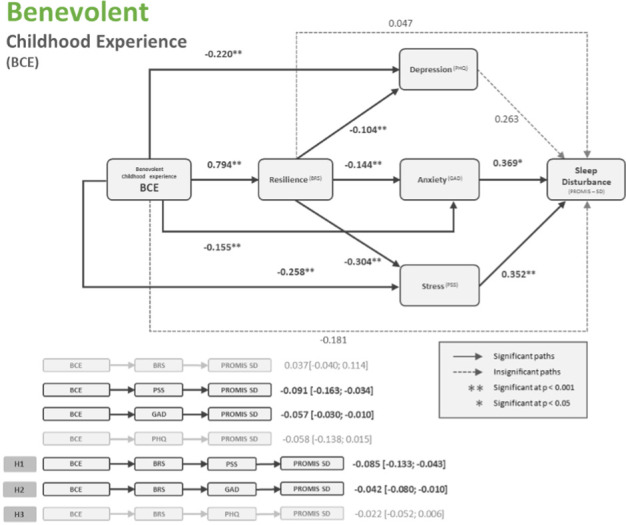
Flow diagram of serial–parallel mediation model with BCE as predictor variable.

When replacing the ACE variable with BCE in this model, very similar outcomes were found. See Table 5. Similar to the ACE model, in the model with BCE as the main predictor, depression was not shown to be a significant predictor of sleep disturbance, hence, it could not play a role in the mediation effects. See Fig 3 for flow diagram.

**Table 5:** Serial–parallel mediation model with BCE as predictor variable. Abbreviations: BCE = Benevolent childhood experience, BRS = Resilience, PHQ = Depression, GAD = Anxiety, PSS = Stress, SD (PROMIS-SD) = Sleep disturbance ‐ (Bold numbers: Statistically significant). Total effect model, direct effects and indirect effects are reported.

*Model*: *Benevolent Childhood experience (BCE) as predictor variable*							
**Total Effect Model**		**b**	**t**	**p**	**95% CI**		**Conclusion**
**BCE ‐ SD**		**-0.498**	**-5.502**	**< 0.001**	**-0.677**	**-0.32**	
*R*^*2*^ *= 0*.*108 | F (399) = 9*.*641 | p < 0*.*001*							
**No.**	**Direct effects**	**b**	**t**	**p**	**95% CI**		
**BCE ‐ BRS**		**0.794**	**7.713**	**< 0.001**	**0.592**	**0.997**	ü
BCE ‐ SD		-0.181	-1.939	0.053	-0.364	0.003	û
**BCE ‐ PHQ**		**-0.220**	**-6.346**	**< 0.001**	**-0.287**	**-0.151**	ü
**BCE ‐ GAD**		**-0.155**	**-4.098**	**< 0.001**	**-0.230**	**-0.081**	ü
**BCE ‐ PSS**		**-0.258**	**-3.937**	**< 0.001**	**-0.387**	**-0.129**	ü
BRS ‐ SD		0.047	1.017	0.310	-0.044	0.137	û
**BRS ‐ PHQ**		**-0.104**	**-6.612**	**< 0.001**	**-0.134**	**-0.073**	ü
**BRS ‐ GAD**		**-0.144**	**-8.389**	**< 0.001**	**-0.178**	**-0.110**	ü
**BRS ‐ PSS**		**-0.304**	**-10.227**	**< 0.001**	**-0.362**	**-0.246**	ü
PHQ ‐ SD		0.263	1.674	0.095	-0.046	0.572	û
**GAD ‐ SD**		**0.369**	**2.643**	**0.008**	**0.095**	**0.643**	ü
**PSS ‐ SD**		**0.352**	**4.455**	**< 0.001**	**0.196**	**0.507**	ü
**No.**	**Indirect effects**	**b**	**Boot SE**		**Boot 95% CI**		
BCE ‐ BRS ‐ SD		0.037	0.039		-0.040	0.114	û
**BCE ‐ PSS ‐ SD**		**-0.091**	**0.033**		**-0.163**	**-0.034**	ü
**BCE ‐ GAD ‐ SD**		**-0.057**	**0.030**		**-0.126**	**-0.010**	ü
BCE ‐ PHQ ‐ SD		-0.058	0.038		-0.138	0.015	û
**H1**	**BCE ‐ BRS ‐ PSS ‐ SD**	**-0.085**	**0.023**		**-0.133**	**-0.043**	ü
**H2**	**BCE ‐ BRS ‐ GAD ‐ SD**	**-0.042**	**0.018**		**-0.080**	**-0.010**	ü
**H3**	BCE ‐ BRS ‐ PHQ ‐ SD	-0.022	0.014		-0.052	0.005	û

**Fig 4:**
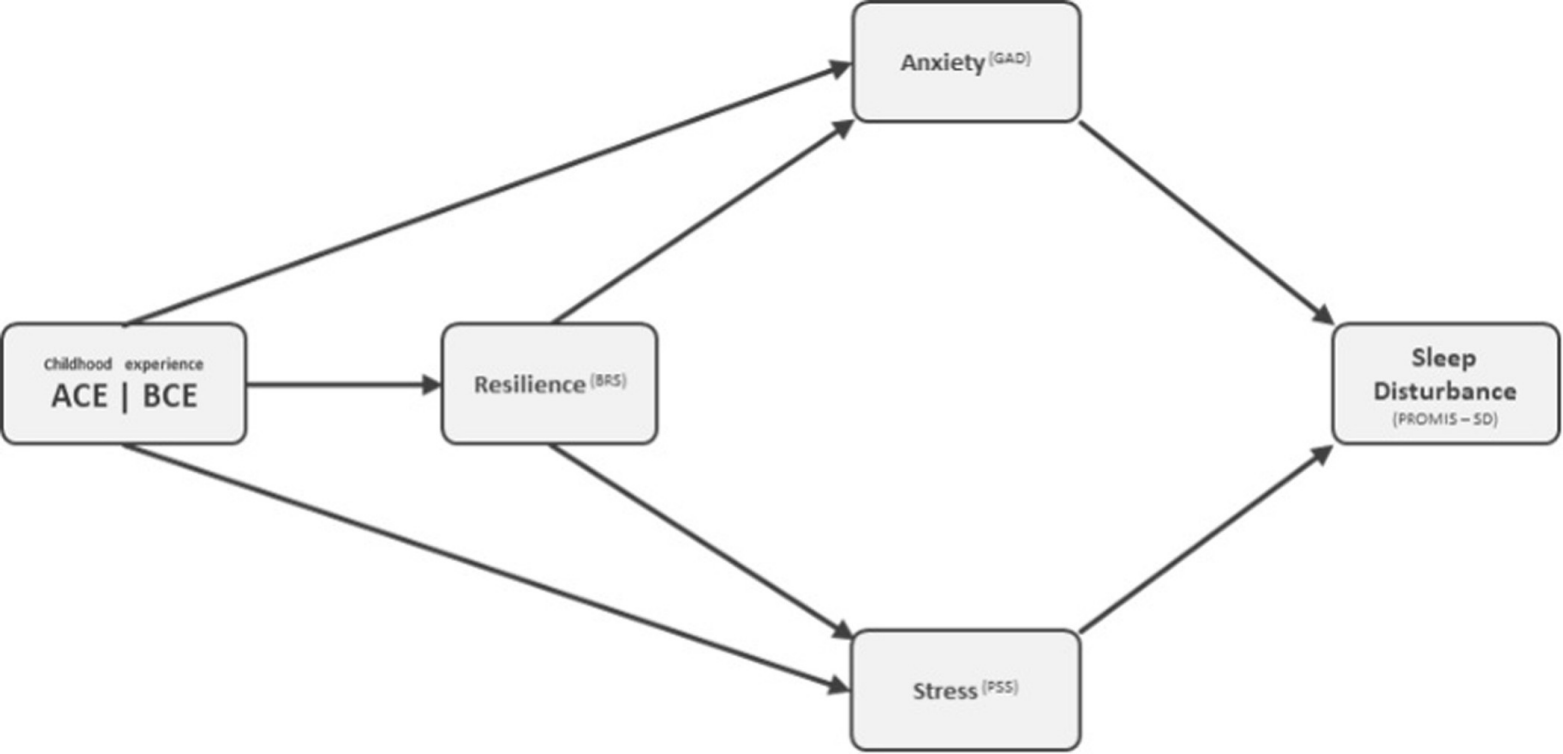
Summary diagram of serial–parallel mediation model for predicting sleep disturbance.

After exploring the relationships between ACE/BCE and mental health measures, resilience and sleep disturbance, a finalized version of the serial-parallel mediation model can be proposed, where depression is removed from the model. In addition, resilience and ACE/BCE are no longer directly connected to sleep disturbance (Fig 4).

## 5. Discussion

This study evaluated the interplay of childhood experiences with resilience, mental health issues and sleep disturbances. This was done by creating a conceptual model in which resilience and mental health issues were hypothesised to mediate the impact of childhood experience on the onset of sleep problems in a serial-parallel way while controlling for age, country of residence, education and ethnicity. Furthermore, participants were asked to indicate whether they had undergone or sought treatment for health conditions to prevent such outcomes from further moderating the impact of ACEs on sleep disturbance levels through the development of better coping methods which has been shown to result in lower levels of stress or anxiety [28].

The current study did not show that depression had a direct effect on sleep but rather the effect of ACEs on sleep disturbances were found to be fully mediated by psychological resilience, anxiety and stress. Moreover, gender was shown not to have any significant impact on any study measure including ACEs. This finding is in contrast with several prior studies conducted on ACEs which suggested that females frequently self-report substantially higher ACEs [29, 30] One possible reason could be due to sample bias in terms of small number of male participants in this study, though at least 25% reported greater than four ACEs increasing up to 30% in Middle Eastern countries. This suggests that ACEs are commonly encountered in females.

Whilst the mediating effect of depression on sleep outcome was noted in other studies for example by Park et al. [31] looking at 737 adolescent students of whom 7% experienced an ACE, and also in a systemic review of 30 published studies by Kajepeeta et al. [7], it may be that this study was limited by its sample size in comparison. Nevertheless, both of these studies in keeping with this study found that anxiety did at least partially mediate the sleep response.

When ACEs were replaced by BCEs as a predictor variable in the conceptual model, a similar finding emerged wherein BCEs was inversely associated with sleep disturbances, and that this effect was mediated by resilience, anxiety and stress. BCEs strongly predicted the psychological resilience of participants. This is consistent with a cross-sectional study conducted by Bethell et al. [32] where in a state-wide sample of Wisconsin (USA) adults who reported higher BCE scores manifested greater general health, which strengthens psychological resilience and moderation of anxiety and stress.

This study has also highlighted the importance of psychological resilience as a predictor of mental health, and that high resiliency improves the quality of sleep via reduced anxiety and stress. This result was also consistent with the findings of a study conducted by Cai et al. [12], who tested a mediation model on 196 Chinese elders with visual and physical conditions who resided in nursing homes. The model used resilience as the predictor variable, perceived stress as the mediator, and sleep quality as the outcome variable. The study demonstrated that higher resilience among Chinese elderly predicts better sleep quality by reducing the perceived stress. The parallel mediation model from our study provides further insight into how resilience might be impacting sleep quality indirectly through stress and anxiety. The detrimental effect of perceived stress and the protective function of resilience on sleep quality was also confirmed in another study by Hrozanova et al. [11].

The findings of this study support these studies in that psychological resilience is an important predictor of mental health, and high resiliency improves the quality of sleep via reduced anxiety and stress. Cai *et al*. [12] tested a mediation model with resilience as the predictor variable, perceived stress as the mediator and sleep quality as the outcome variable. The authors demonstrated that higher resilience among Chinese elderly predicts better sleep quality by reducing the perceived stress [12]. However, unlike Cai *et al*. [12], the results of the current study could not confirm a direct effect of resilience on sleep quality, and its effects were fully mediated by stress and anxiety. The detrimental effect of perceived stress and the protective function of resilience on sleep quality was also confirmed in another study by Hrozanova et al. [11].

In our study participants from the UK made up nearly 50% of the sample size with 53–67% reported no ACEs and 1–14% reporting ≥ 4 ACEs. This is in contrast to Middle Eastern participants who reported much lower lack of ACE prevalence (18%) and much higher (32%) of ≥ 4 ACE prevalence, suggesting that UK participants may be generally exposed to fewer ACE types than participants residing in Middle east. This is in keeping with the meta-analysis by Hughes et al. [33], who looked at reported ACEs from a variety of countries and found a slightly higher rate in Middle Eastern compared to western countries (mean number of 12 compared to an overall mean of 9 ACEs).

The authors acknowledge noteworthy limitations of this study. This was a cross-sectional study looking at associations rather than causation. In addition, the survey was sent to the respondents online, which could disproportionately exclude the individuals who either did not have access to or felt uncomfortable using the internet. Given that this study was conducted by self-reported questionnaires, the authors acknowledge that the results may be prone to response bias. Thus, future longitudinal studies are needed to confirm the current findings.

Moreover, the sample of the study included mainly highly educated females from high-income countries, and future studies are needed to generalize the results to different populations. Despite such limitations, the current study can have important implications for health care policies, contributing to the knowledge about the impact of ACEs on sleep disturbances and the potential positive role that psychological resilience can play to reduce mental health issues and mitigate sleep problems.

## 6. Conclusion

The results of this study underlines the detrimental effects of adverse childhood experiences on mental well-being, and highlights the significance of psychological resilience in moderating the negative effects of stress on sleep quality. The final proposed model concludes that the two predictor variables (ACE/BCE) indirectly affect the degree of sleep disturbances through the three mediators (resilience, stress and anxiety), whereby high ACE predicts lower sleep quality and high BCE predicts higher sleep quality. Hence, the development of comprehensive health guidelines that incorporate aspects of resilience, anxiety and stress is essential in improving sleep quality for those who experienced ACEs.
